# Gait and heart rate: do they measure trait or state physical fatigue in people with multiple sclerosis?

**DOI:** 10.1007/s00415-024-12339-8

**Published:** 2024-05-02

**Authors:** Irina Galperin, David Buzaglo, Eran Gazit, Nathaniel Shimoni, Raz Tamir, Keren Regev, Arnon Karni, Jeffrey M. Hausdorff

**Affiliations:** 1 Center for the Study of Movement, Cognition and Mobility, Neurological Institute, Tel Aviv Sourasky Medical Center, Tel Aviv, Israel; 2 Department of General Medicine, Faculty of Medical & Health Sciences, Tel Aviv University, Tel Aviv, Israel; 3 Department of Physical Therapy, Faculty of Medical & Health Sciences, Tel Aviv University, Tel Aviv, Israel; 4 Owlytics Healthcare Ltd., Ramat-Gan, Israel; 5 Department of Information Systems Engineering, Ben-Gurion University of the Negev, Beer-Sheva, Israel; 6 Neuroimmunology and Multiple Sclerosis Unit of the Department of Neurology, Tel Aviv Sourasky Medical Center, Tel Aviv, Israel; 7 Department of Neurology and Neurosurgery, Faculty of Medical & Health Sciences, Tel Aviv University, Tel Aviv, Israel; 8 Sagol School of Neuroscience, Tel Aviv University, Tel Aviv, Israel; 9 Rush Alzheimer’s Disease Center and Department of Orthopedic Surgery, Rush University Medical Center, Chicago, USA

**Keywords:** Multiple sclerosis, State fatigue, Physical fatigue, Gait, Heart rate, Autonomic nervous system

## Abstract

**Background:**

Trait and state physical fatigue (trait-PF and state-PF) negatively impact many people with multiple sclerosis (pwMS) but are challenging symptoms to measure. In this observational study, we explored the role of specific gait and autonomic nervous system (ANS) measures (i.e., heart rate, HR, r–r interval, R–R, HR variability, HRV) in trait-PF and state-PF.

**Methods:**

Forty-eight pwMS [42 ± 1.9 years, 65% female, EDSS 2 (IQR: 0–5.5)] completed the Timed Up and Go test (simple and with dual task, TUG-DT) and the 6-min walk test (6MWT). ANS measures were measured via a POLAR H10 strap. Gait was measured using inertial-measurement units (OPALs, APDM Inc). Trait-PF was evaluated via the Modified Fatigue Impact Scale (MFIS) motor component. State-PF was evaluated via a Visual Analog Scale (VAS) scale before and after the completion of the 6MWT. Multiple linear regression models identified trait-PF and state-PF predictors.

**Results:**

Both HR and gait metrics were associated with trait-PF and state-PF. HRV at rest was associated only with state-PF. In models based on the first 3 min of the 6MWT, double support (%) and cadence explained 47% of the trait-PF variance; % change in R–R explained 43% of the state-PF variance. Models based on resting R–R and TUG-DT explained 39% of the state-PF.

**Discussion:**

These findings demonstrate that specific gait measures better capture trait-PF, while ANS metrics better capture state-PF. To capture both physical fatigue aspects, the first 3 min of the 6MWT are sufficient. Alternatively, TUG-DT and ANS rest metrics can be used for state-PF prediction in pwMS when the 6MWT is not feasible.

**Supplementary Information:**

The online version contains supplementary material available at 10.1007/s00415-024-12339-8.

## Introduction

Fatigue is a hallmark symptom of multiple sclerosis (MS) and affects a significant majority of people with MS (pwMS) [1, 2]. About half of pwMS consider fatigue to be their most debilitating issue [3]. Fatigue in MS is associated with disease severity, cognitive deficits, work capacity, and fall risk [4, 5]. Physical fatigue (PF) is defined as a decrease in muscular strength during moderate activities, such as walking, leading to loss of mobility and daily activity limitations [6]. It is also characterized by a need for rest without necessarily requiring sleep [7]. PF can be caused by direct lesions or by the effects of these lesions on muscles, autonomic functions, and other systems [1, 8]. PF is a complex symptom with physical, cognitive, and psychological contributors and multiple, negative consequences.

To date, fatigue has been primarily assessed through self-report questionnaires such as the Fatigue Severity Scale (FSS) and the Modified Fatigue Impact Scale (MFIS) [9]. These questionnaires measure the cognitive, psychological, and physical components of fatigue and have been commonly used to track changes over time and evaluate the effectiveness of interventions [9, 10]. In general, the physical component of the MFIS reflects the perception of reduced physical energy and the difficulty of initiating and sustaining physical activity in daily life [11].

Although extensive efforts have been made to find effective treatments for fatigue in MS, definitive conclusions on the effectiveness of such treatments remain elusive. One reason could be that subjective questionnaires may not be sensitive enough [10]. Furthermore, there is a lack of standardized and objective tools for assessing physical fatigue and fatigability [12]. Therefore, there is increasing recognition of the need for objective and sensitive tools and measures to assess and monitor different aspects of fatigue in MS [13–15].

Another consideration is that existing measures may not adequately differentiate between trait and state fatigue and fatigability. *Trait fatigue* has been evaluated through questionnaires that estimate the capacity to perform various tasks, while *state fatigue* has been evaluated by the perceived feeling of tiredness following fatiguing tests [16]. In addition, performance fatigability, which reflects changes in objectively measured variables during task performance, provides another measure of fatigue. Consequently, distinct measures are likely required to capture these different aspects of fatigue [13, 16].

Initial attempts have been made to address the gaps in delivering objective measures of different fatigue aspects. Some studies have reported associations between state and trait fatigue and changes in gait metrics (e.g., cadence) during the 6-min walk test (6MWT) [14, 15]. One study, conducted in a small group of pwMS, found that performance fatigability was primarily associated with disability, as assessed by the Expanded Disability Status Scale (EDSS), rather than trait or state fatigue rating scales [13]. However, there has been no systematic evaluation of the relationships between the different aspects of fatigue and objective measures in pwMS, particularly regarding the physical component of trait fatigue.

In addition to gait impairments, pwMS also experience dysregulation of the autonomic nervous system (ANS). This too may contribute to trait and state fatigue progression [17–19]. There is evidence linking ANS activity in response to cognitive effort to state fatigue in MS [20]. Moreover, Flachenecker et al., (2003) observed abnormal autonomic function in response to physical exertion (grip) in pwMS who experienced fatigue, but not in those without fatigue (who did not differ from healthy controls) [21]. These ANS alterations were associated with the physical component of trait fatigue (as measured by the MFIS questionnaire), indicating that trait fatigue can be partially explained by autonomic dysfunction.

To date, no studies have simultaneously evaluated the contribution of gait, heart rate, and ANS activity to the physical component of trait fatigue (trait-PF) and state fatigue (state-PF) in pwMS. To address these gaps, we examined the associations between ANS (e.g., heart rate, r–r intervals, and their variability), gait performance, trait-PF, and state-PF to identify objective markers of those aspects of fatigue in laboratory settings using a 6MWT test in pwMS. Given that dual-task abilities are essential for everyday functioning but are impaired in pwMS and that these abilities might contribute to the perception of fatigue, we also investigated the associations between the Timed Up and Go (TUG) test performance—both with and without a dual-task component—and trait-PF and state-PF.

## Patients and methods

### Participants

In this observational study, subjects were recruited from the Neuroimmunology and Multiple Sclerosis Unit, Tel Aviv Sourasky Medical Center, Israel, using a convenient sampling method between March 2021 and January 2022. We included participants who met the following criteria: (a) physician-confirmed diagnosis of RRMS according to McDonald criteria 2017 [22], with EDSS 0–5.5, (b) between 18–65 years old, (c) ambulatory (walking aid allowed), (d) community living, and (e) free of relapse in the past 30 days. We excluded participants if they: (a) could not walk for 6 min, (b) had another neurological condition, unstable cardiovascular disease, or any acute rheumatic, surgical, or orthopedic problems that may interfere with walking, (c) were currently pregnant, or d) had a pacemaker. All patients provided written informed consent before any study procedure was performed.

### The experimental procedure

After obtaining written informed consent, basic demographic characteristics were collected (e.g., age, sex, height, weight, disability status). Cognition was accessed using the Montreal Cognitive Assessment (MoCA Test). The participants then completed several questionnaires: MFIS to report fatigue, the International Physical Activity Questionnaires (IPAQ) to assess the amount of weekly physical activity, and the Hospital Anxiety and Depression Scale (HADS) to assess anxiety and mood alterations. Then the participants performed a Timed Up and Go test, with (TUG-DT), and without (TUG) an additional task [23]. They rested lying down for 5 min followed by 5 min sitting in a quiet room. After the rest period, the participants performed the 6MWT. Subjects were asked to cover as much distance as possible while walking back and forth over a 25-m path, according to accepted guidelines (e.g., informed when completed half of the test) [24]. Finally, the participants recovered while seated for 5 min. State-PF before and after the 6MWT was reported using a visual analog score (VAS). Heart rate (HR), r–r intervals (R–R), and heart rate variability (std of the R–R, SDRR) were measured throughout the study using a Polar strip. Gait performance was recorded during the 6MWT via wearable sensors.

### Timed up and go test

Since trait physical fatigue (as reflected in the MFIS questionnaire) and perceived state fatigue has the potential to influence everyday functions, and since everyday functions are usually multifunctional, we added a functional Timed Up and Go (TUG) test with (TUG-DT) and without an additional task. The TUG-DT included a widely used cognitive task that participants were asked to perform at the same time that they performed the TUG. The cognitive task was counting backwards subtracting 3 from a given number [23]. The simultaneous performance of serial 3 subtractions depends on the ability to divide attention and challenges attentional resources. It is considered a task of moderate difficulty.

### Trait physical fatigue (trait-PF) assessment

Trait fatigue was assessed using the MFIS [25]. The physical subscale is computed based on a subset of items of the MFIS questionnaire and scores range from 0–36 (MFIS_phys_). Higher scores indicate a greater impact of fatigue on daily activities. This questionnaire is considered the gold standard for assessing overall trait fatigue and the physical aspect of it, as well as other dimensions.

### State physical fatigue (state-PF) assessment

**Fig. 1:**
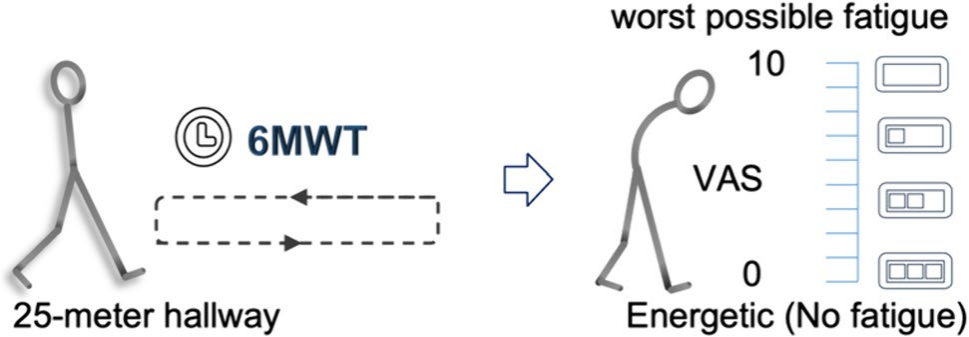
Visualization of state-PF evaluation. Subjects reported their state-PF using the Visual Analog Scale (VAS) before and after performing the 6MWT. 0 denotes no perceived fatigue and 10 denotes maximal perceived fatigue

State fatigue was evaluated via a VAS scale (0–10) (Fig. 1). VAS “0” indicated no fatigue, and “10” indicated the most severe fatigue. Subjects were asked to report their perceived physical fatigue at the end of the 6MWT.

### Collection of the autonomic nervous system (ANS) data

ANS function was evaluated using a chest strap (H10 Polar) that measured the ECG signal [26]. HR and R–R (the time elapsed between two successive R waves of the QRS signal on the electrocardiogram (and its closely related reciprocal, the HR) were determined using a smartphone application (app) HRV Expert (CardioMood). The tool was previously used in research to measure HRV [27]. HR and HRV were collected during rest and during the 6MWT. The collected data were stored on a dedicated smartphone. Subsequently, median values of HR and R–R were extracted in each minute of the 6MWT. The standard deviation of the R–R (SDRR) was calculated using MALTAB (MathWorks, Natick, MA, USA) software.

### Gait

Spatio-temporal gait metrics were collected using OPALs (Opal, APDM, Portland, OR, USA) wearable sensors fixed to the lower back and ankles of the participants [28]. Offline, gait data were transferred to a personal computer for processing using MATLAB (MathWorks, Natick, MA, USA) software. We used only the straight-line walking gait (turns were removed). For each minute of the 6MWT, spatial–temporal features of gait were extracted. We based the gait parameter selection on five independent domains (i.e., pace, rhythm, postural control, variability, and asymmetry) [29]. To minimize redundancy, we used one or two representative gait parameters of each domain. We also calculated the area under the curve between the 3rd, the 6th, and the remaining minutes of walking, referred to below as the “Cadence area”. A more detailed description of the cadence area can be found in the Supplementary Methods.

Prior to conducting the statistical analysis, we computed the maximum, minimum, and range of each gait and ANS metric of the first 3 min, and the entire 6 min of the test. This helped us capture any performance fluctuations that could be attributed to fatigue.

### Statistical analysis

All statistical analyses were conducted using Prism 9.5.1 (2023) (GraphPad Software, San Diego, California USA) or SPSS (version 27.0, IBM, Armonk, NY, USA). The normality of the data was assessed using Shapiro–Wilk test. Repeated measures ANOVA (with the Sidak test to control for multiple comparisons), the Friedman test, and the Wilcoxon signed rank test were used to evaluate gait and ANS changes that occurred during the first 3 min and all 6 min of the 6MWT.

As a first step in identifying the independent predictors of trait-PF and state-PF, we examined the correlations between trait-PF, state-PF, and the metrics within each group (ANS and gait) using Pearson and Spearman’s correlation tests. If the variables entered into the analysis had a normal distribution, we used Pearson analysis. Otherwise, we used Spearman’s method. Within each group of parameters, we selected the parameters that were most strongly and significantly (*p* < 0.05) associated with the dependent variable (trait-PF or state-PF) while removing measures that were strongly correlated with one another (*r* > 0.8) for the multivariable linear regression models.

Regarding gait variables, we first selected the representative of each gait domain (e.g., pace, rhythm) [30] using the approach mentioned above. Then the process was applied to mean/max/min values within the representatives of each metrics group (gait and ANS) that later were entered into the regression analysis. We performed a series of hierarchical regression models with trait-PF or state-PF as the dependent variable while the independent variables were entered into the analysis using blocks. The first block consisted of age, sex, weight, height, and EDSS, as these have the potential to influence trait and state physical fatigue, gait, and ANS metrics. The previously selected representatives of each metrics group, gait, or/and ANS, were entered as a second block using a forward stepwise method. A variable was entered into the model if its F ANOVA value’s significance level was less than 0.05.

## Results

Forty-eight people with pwMS were studied. Demographics and disease-related characteristics are presented in Table 1. Participants had varying levels of disease severity (EDSS 2 [0–5.5]) and preserved cognition (MoCa 27.49 ± 2.30). Most patients were taking disease-related medication (87%) and reported moderate mood disorders (HADS 11.11 ± 6.92). A minority, 0.8%, were taking cardiovascular medications. A more detailed medication list can be found in the Supplementary Results Table 1.

**Table 1:** Demographics, disease-related, and other measures

Variable	(*n* = 48)
Age (years)	47.9 $\pm$ 11.23
Female (%)	65.0
EDSS	2 (IQR: 0–5.5)
MoCA	27.49 $\pm$ 2.30
IPAQ (met)	2662.5 $\pm$ 3231.6
HADS	11.11 $\pm 6.92$
Disease-related medications (%)	87.5
Cardiovascular medications (%)	0.8
MFIS total	31.93 $\pm$ 16.57
MFIS physical	16.12 $\pm$ 8.96
MFIS cognitive	12.74 $\pm$ 8.03
MFIS psychological	2.57 $\pm$ 2.16
TUG	9.55 $\pm$ 2.71
TUG-DT	10.80 $\pm$ 2.96
Gait speed (m/sec)*	1.28 $\pm$ 0.34
Cadence (steps/min)*	114.39 $\pm$ 15.90
Double support (%)*	24.96 $\pm$ 5.54
HR (bpm) at rest**	74.22 $\pm$ 11.17
R-R (ms) at rest**	827.15 $\pm$ 129.29
SDRR (ms) at rest**	51.20 $\pm$ 28.50
VAS before 6MWT	2 (0–9)
VAS after 6MWT	4 (0–10)
Distance walked in 6MWT [m]	456 $\pm$ 141

Data are presented as mean (SD) or median (IQR).* EDSS* expanded disability status scale. *MoCA* montreal cognitive assessment. *IPAQ* physical activity questionnaire (MET minutes a week), *HADS* hospital anxiety and depression scale. *TUG* Timed Up and Go test, *TUG-DT* TUG while performing a dual task, serial 3 subtractions. *MFIS*—the modified fatigue impact scale. *HR* heart rate. *R-R r-r* intervals. *SDRR* SD of R-R. *VAS* visual analog scale. 6MWT 6-min walk test

*The values reported are mean $\pm {\text{s}}.{\text{d}}$ of the first minute of the 6MWT. ** measured while subjects were seated for 5 min before performing the 6MWT

**Fig. 2:**
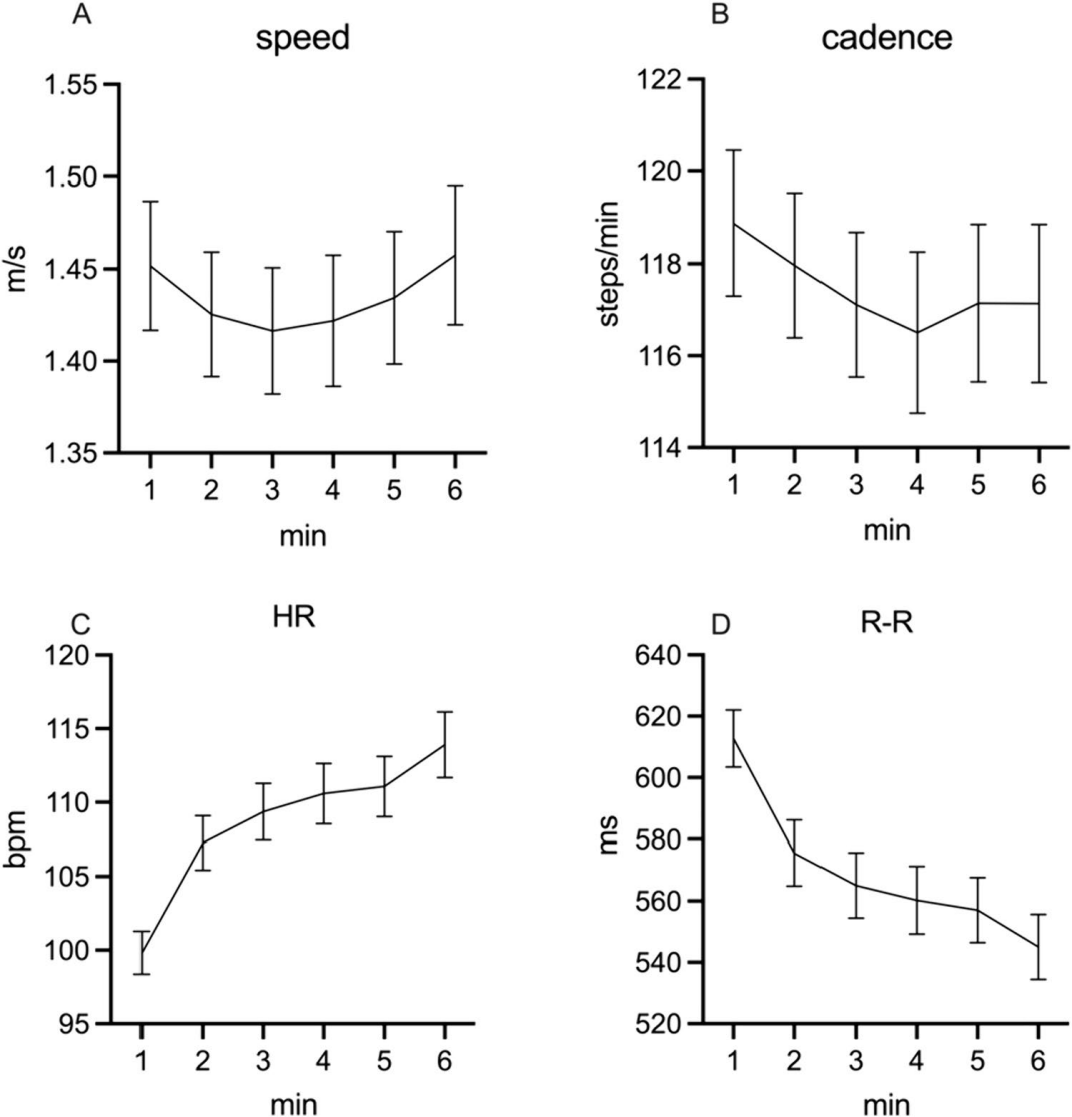
Gait and ANS metrics at each minute of the 6MWT. The *x*-axis represents time (min). The *y*-axis represents mean and error (SEM) of gait and ANS metrics across the minutes of the 6MWT. **A** speed (m/s); **B** cadence (steps/min); **C** HR: heart rate (bpm); **D** R–R: r–r intervals (ms). 6MWT: 6-min walk test. As expected, the R–R changes roughly mirror the changes in HR

Figure 2 shows that during the 6MWT, gait speed and cadence changed in a U-like fashion, reaching their lowest values during the third and fourth minutes of the 6MWT. In contrast, HR continued to rise, and the R–R continued to decline throughout the 6MWT. Supplementary Table 2 details the changes in time for the gait and ANS measures. For example, HR rose significantly during the 6MWT (*F* = 104.537, *p* < 0.001), while other measures (e.g., gait asymmetry) changed only slightly (non-significantly). The VAS score increased from 2 [95 CI 2.05–3.5] as reported at baseline (before performing the 6-min walk) to 4 [95 CI 3.07–5.05], as reported after the 6MWT (*p* < 0.001 according to the Wilcoxon test) indicating that the 6MWT may be considered as fatigue provoking test.

### Associations between the trait-PF, gait, and ANS metrics measured during the 6MWT

**Fig. 3:**
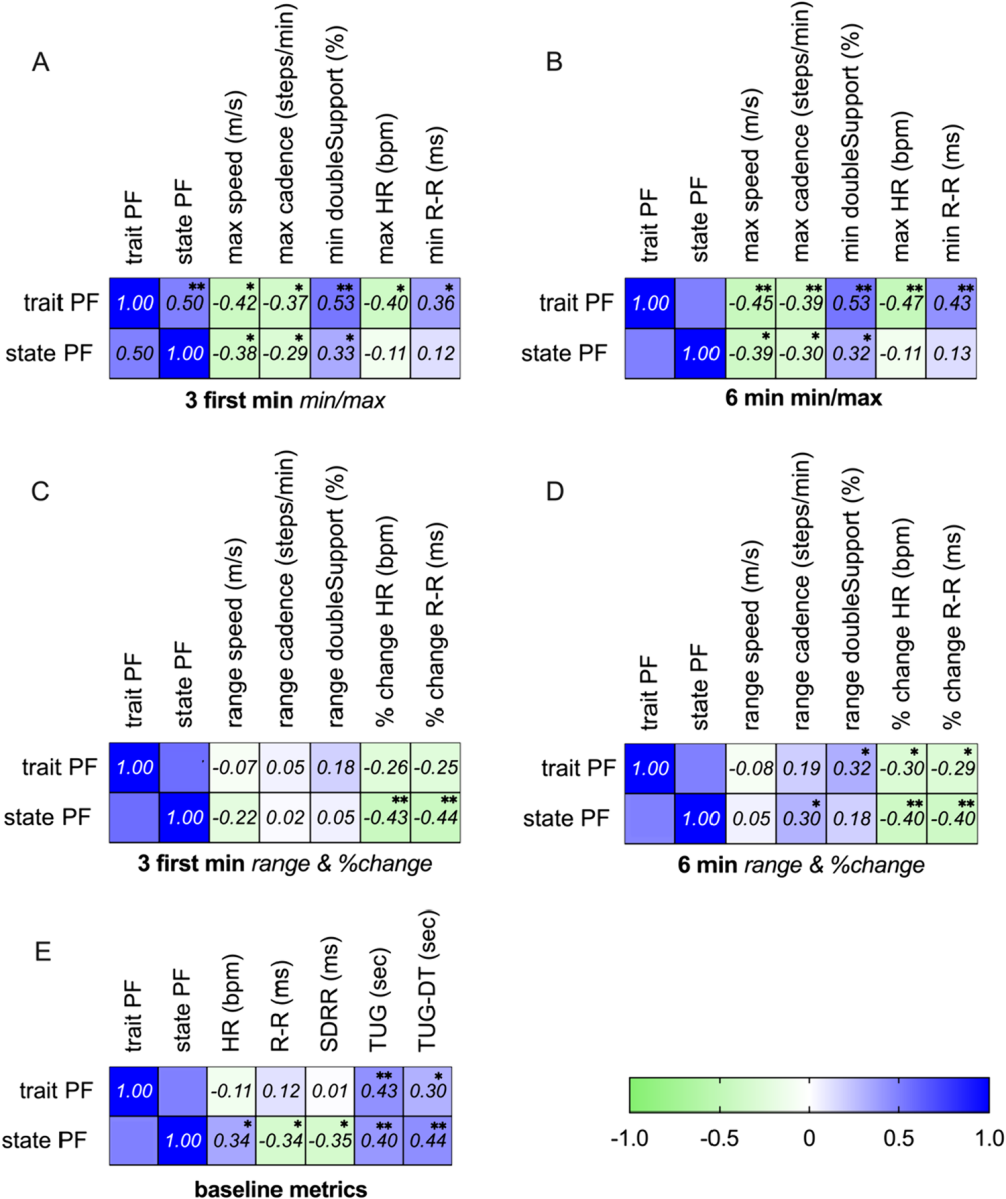
Associations between trait-PF, state-PF, and objective measures collected during 6MWT. A type of a heatmap. The colors represent the strength of the correlations between trait-PF, state-PF and the following objective measures: **A** min and max values of gait and ANS metrics measured during the first 3 min of the 6MWT, **B** min and max values of gait and ANS metrics measured during the entire 6MWT, **C** range of the gait and ANS metrics measured during the first 3 min of the 6MWT, **D** range of the gait and ANS metrics measured during the entire 6MWT, **E** metrics at baseline (before performing the 6MWT). *Statistical significance of p value < 0.05. **Statistical significance of p value < 0.01. HR: heart rate (bpm), R–R: r–r intervals (ms). SDRR: the standard deviation of R–R, a representative of heart rate variability (ms). TUG: Timed Up and Go test, TUG-DT: TUG while performing a dual task, serial 3 subtractions

The MFIS _physical_ scores were significantly associated with objective metrics (i.e., gait and HR collected at the time points of the experiment except for the SDRR), as shown in Fig. 3**.** Linear regression models applied to evaluate the strongest trait-PF predictors adjusted for subject characteristics are presented in Table 2. Table 2A presents the models based on the objective metrics collected during the first 3 min of the 6MWT, while Table 2B presents the models based on the objective metrics collected during all 6 min of the test. The rationale of dividing the test into two is that after 3 min (half of the test), the subjects were told “you have now completed 3 min of the test, another 3 min remain”. The subjects tend to increase their gait speed at this point (see Fig. 2 and Supplementary Results Table 2). In contrast, changes in the objective metrics during the first 3 min may indicate “natural” fatigability, which is not masked by the effort of the last 3 min.

**Table 2:** Trait-PF and state-PF explained by objective metrics of gait and ANS collected during the first half and all minutes of the 6MWT

	A. Models of 3 first minutes					B. Models of 6 full minutes				
		*r*^2^	Predictors	St. Beta	*P* value		*r*^2^	Predictors	St. Beta	*P* value
trait-PF	1	.390**	Min double support [%]	0.5	<0.001	1	0.376**	Min double support [%]	0.436	0.004
			Cadence range	0.32	0.013					
	2	.467**	Cadence area	0.292	0.021	2	0.463*	Min double support [%]	0.395	0.025
			Min double support [%]	0.472	<0.001			change in HR[%] * change in speed [%]	− 0.359	0.03

	C. Models of 3 first minutes					D. Models of 6 full minutes				
		*r*^2^	Predictors	St. Beta	*P* value		*r*^2^	Predictors	St. Beta	*P* value
State-PF	1	.412**	R–R range 3 min	− 0.545	0.001	1	0.348*	R–R range	− 0.508	0.001
			Cadence range							
	2	.426**	HR first 2 mins Change [%]	0.562	0.001	2	0.403**	R–R range	-0.537	0.001
			Cadence range					Cadence range		

*p value of the model < 0.05 **p value of the model < 0.001. All models adjusted to age, sex, weight, height, and EDSS. The variables were entered into the regression using the forward method in blocks: demographics in the first block and then the rest. St: standardized. **A**. Trait-PF explained by metrics collected during the first 3 min of the 6MWT** B**. Trait-PF explained by metrics collected during entire 6 min of the 6MWT. **C**. State-PF explained by metrics collected during the first 3 min of the 6MWT **D**. State-PF explained by metrics collected during entire 6 min of the 6MWT. HR: heart rate (bpm), R–R: r–r intervals (ms)

We also explored gait variables from other gait domains, e.g., variability and asymmetry (Supplementary Results Table 2). These measures were not included in further analysis due to irregular distributions and a lack of high correlation with trait-PF or state-PF (step regularity: *r*_*s*_ = − 0.210, *p* = 0.87 and − 0.130, *p* = 0.377, gait asymmetry *r*_*s*_ = − 0.250, *p* = 0.071 and 0.004, *p* = 0.977, respectively).

As shown in Fig. 2, the gait and ANS metrics during the 6MWT show a reduction in cadence simultaneous with a HR increase and R–R decrease. Following these findings, we calculated the area under the curve for these metrics. We specifically looked at the area under the curve of the cadence. The maximal speed and cadence from the first 3 min of the 6MWT explained about 26% of the trait-PF variance. Additional information on regression models, including metrics that did not enter the final models, is detailed in Supplementary Results. In a combined model, double support and cadence area under the curve (adjusted) explained about 47% of trait-PF variance. ANS metrics did not contribute to the combined model of the first 3 min but independently explained some of the trait-PF variance (up to 40%) (see Supplementary Results Table 7).

Information collected during the second half of the 6MWT did not contribute to the trait-PF variance explained by metrics collected during the first 3 min (see Table 2B). Yet, models based on entire 6 min of the 6MWT showed that HR–gait speed interaction played a role in the final model alongside minimal double support, indicating that ANS function measured during more prolonged efforts may reflect trait-PF.

### Associations between state-PF, gait, and ANS metrics measured during the 6MWT

Associations between perceived fatigue (state-PF) reported at the end of the 6MWT and objectively collected metrics are shown in Fig. 3. ANS and gait measures showed a mild to moderate association with state-PF. The change in HR and R–R during the first half of the 6MWT test, followed by TUG-DT, showed the most significant associations with state-PF.

The regression models used to identify the best state-PF predictors (adjusted to demographics) are summarized in Table 2C, D. Table 2C presents the models based on the metrics collected during the first 3 min, while Table 2D presents the models based on the metrics collected during all 6 min of the 6MWT. EDSS explained 11.7% of the state-PF (St. Beta = 0.341, *p* = 0.013). The final model based on the first 3 min explained 42.6% of the state-PF variance, mainly due to the changes in ANS metrics. As presented in Table 2C, the best predictors of state-PF were the range or the percent change in R–R as compared to these metrics at rest, before the 6MWT.

Models based on all 6 min of the test did not contribute any additional explained variance (see Table 2D). Double support (%) explained only a small portion of the state-PF variance (see Supplementary Results Table 9). Notably, gait speed values measured during the first 2, 3, or all 6 min did not contribute to the value of state-PF. However, the cadence range across the 6MWT did contribute when combined with the change in the R–R.

**Fig. 4:**
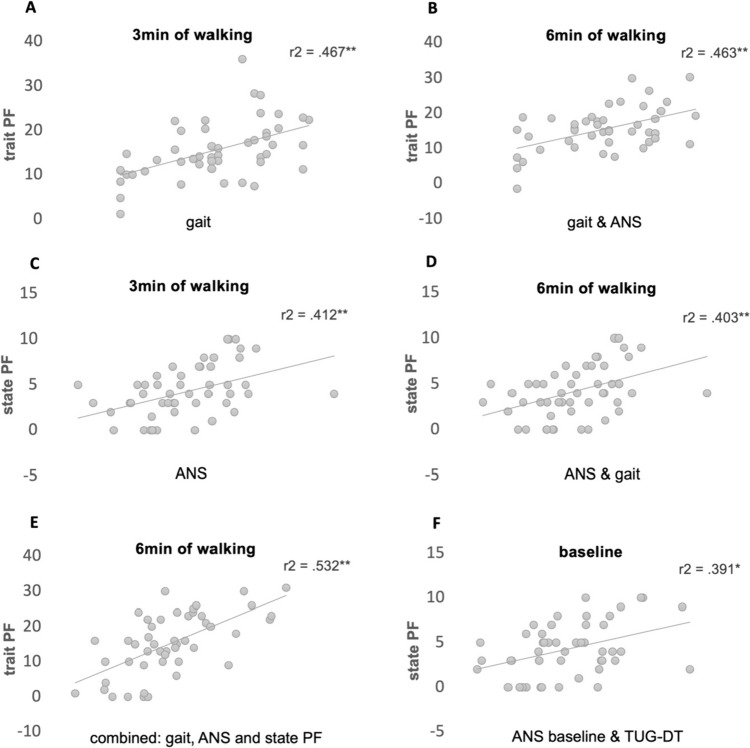
Predicted estimates of trait-PF and state-PF (final models) of 3 and 6 min of the 6MWT.The *y*-axis shows the model’s estimate of trait-PF or state-PF. The *x*-axis shows the factors included in the prediction model.** A** Linear regression combined model with trait-PF (*y*-axis) as the dependent outcome, predicted by objective metrics from the first half of the 6MWT. **B** Linear regression with trait-PF (*y*-axis) as the dependent outcome, predicted by objective metrics from the entire 6MWT. **C** Linear regression combined model with state-PF (*y*-axis) as the dependent outcome, predicted by objective metrics collected during the first half of the 6MWT. **D** Linear regression with state-PF (*y*-axis) as the dependent outcome, predicted by objective metrics collected during all minutes of the 6MWT. **E** Linear regression with trait-PF (*y*-axis) as the dependent outcome, predicted by objective metrics collected during the 6MWT, plus state-PF. **F** Linear regression combined model with state-PF (*y*-axis) as the dependent outcome predicted by objective metrics collected prior to the 6MWT; ANS (autonomic nervous system) baseline R–R (mean of 2 last min of rest sited, before performing the 6-min walk), and TUG-DT (Timed Up and Go test,TUG, while performing a dual task, serial 3 subtractions). All variables were entered into the regression models using the forward method with blocks. Variables that were entered into the first block were: (1) age, height (cm), weight (kg), gender, and EDSS. The second block contained other variables, as detailed in Tables 1 and 2. *Indicates *p* < 0.05, **p < 0.001

Interestingly, TUG-DT alone (adjusted) explained 24% of the state-PF. Based on these findings, we decided to use a model that does not require the 6MWT. The model included TUG-DT, R–R (measured at rest before the 6MWT). Adjusted to age and EDSS, it explained 39% of the state-PF (see Fig. 4F). Supplementary Results Tables 5 and 6 detail the baseline predictors (the metrics collected before the 6-min walk) of state-PF and trait-PF. HRV (as measured by SDRR at rest, before the 6-min walk) showed mild but significant association with state-PF (*r*_*s*_ = − 0.35, *p* = 0.008), yet contributed less than the R–R, when entered into the regression model. Although trait-PF and state-PF correlate moderately (*r*_*s*_ = 0.503, *p* < 0.001), different objective metrics explained trait-PF and state-PF. The scatterplots of the main models explaining the trait-PF and the state-PF are presented in Fig. 4.

## Discussion

In this study, we found a moderate association between the trait-PF (according to the physical component of the MFIS questionnaire) and state-PF (perceived fatigue reported by VAS at the end of the fatigability-provoking test, 6MWT) in individuals with MS. We observed that gait metrics and ANS measures were both associated with trait-PF and state-PF. Gait metrics explained a significant portion of the variance of trait-PF (47%), while ANS metrics explained a roughly similar portion of the variance of state-PF (43%).

Consistent with previous findings [31], we observed a deceleration in spatio-temporal gait parameters during the initial 3 min of the 6MWT, likely reflecting physical fatigability. Our findings indicate that the first 3 min of the test provided sufficient information and were as informative as the full 6 min in terms of trait-PF. Specifically, double support percent, a measure that has previously been linked to disease severity [32] and perceived fatigue [33], was most strongly associated with trait-PF. Higher values of minimal double support (%) in individuals with more physical fatigue indicate that these individuals spent more time with both feet on the ground during the gait cycle, even during the least-fatigued periods of the 6MWT. The prolonged double support could be indicative of one’s attempt to compensate for balance difficulties, which occur due to emerging muscle tiredness and a decreased range of motion in the lower extremities [34], ultimately leading to poorer balance.

In contrast to gait metrics, heart rate (HR) continued to rise, while the R–R interval declined throughout the 6MWT. The observed rising cardiac effort, together with an increased feeling of exertion reported at the end of the test, suggest that the 6MWT test is fatigability-provoking in pwMS. This observation aligns with previous reports of reduced maximal aerobic capacity in people with MS-related fatigue [35] and previous evidence on the association between self-reported fatigue and ANS dysfunction in MS [17, 36]. Here we provide new evidence based on objective measures. In our study, maximal HR and R–R were associated with trait-PF, and the interaction between HR and gait speed contributed to the model of the full 6 min of the 6MWT, highlighting the association between physical fatigue, gait, and ANS changes during prolonged effort.

Regarding state-PF, the last 3 min of the test did not provide additional information beyond the first 3 min. The strongest predictor of state-PF was the change in R–R interval during the first 3 min of the test, explaining 43% of its variance. Similar to a recent publication that evaluated the impact of the rate of perceived exertion on gait by Theunissen et al. [37], we confirmed increased exertion (i.e., what we refer to as state fatigue) following the 6MWT. Yet, the study by Theunissen et al., (2023) primarily observed changes in walking speed without other alterations in the cost of other metrics of walking or muscle activation, suggesting that perceived exertion increases without a major increase in objective physical exertion measures. Here we extend those findings by utilizing heart rate variability and detailed gait analysis, offering a broader perspective on the physiological markers of fatigue. Our results support the notion that objective markers collected through the 6MWT can account for perceived exertion.

We also found that the rest r–r intervals and the Timed Up and Go test with a dual-task component were almost as effective in predicting state-PF (39%) as the first 3 min of the 6MWT. These results suggest that combined with ANS measures, relatively short functional tests in the laboratory could be alternatives for evaluating state fatigue when a prolonged effort is not feasible and correspond with that reported by Ibrahim et al. (2022) [14]. TUG and TUG-DT are routinely used as part of a dynamic balance and mobility battery to assess physical functioning under divided attention. However, we did not find previous reports on the association between TUG-DT and state-PF in pwMS. Only weak associations between TUG (without dual task) and trait-PF were reported [38]. Our findings support the hypothesis that motor–cognitive interference may play a role in self-perceived physical fatigue. In addition, our results extend previous knowledge regarding the connection between cognitive performance, fatigue [39], and ANS function [20] to include the association with state-PF. Finally, our results emphasize the need for further investigation into the role of divided attention in state and trait physical fatigue and the association with ANS in pwMS.

Some limitations of this study include the lack of a group of age- and sex-matched healthy controls. In addition, the study included individuals with a wide range of disease severity. Although we adjusted all regression models to EDSS, future studies with larger groups of subjects should investigate these objective trait and state fatigue markers in different EDSS levels (to examine if the relationships may depend on disease stage). Including additional factors related to fatigue in MS, such as pain, muscle force, joint mobility, and smoking, in future studies would also be informative [40, 41]. Furthermore, systemic medications and those that may influence central nervous system function are described in the manuscript. However, due to the size of the cohort, it was not possible to include all potential factors in the analysis. It is also worth noting that our sample was not very impaired in terms of trait fatigue, which may limit the applicability of our findings to more impaired populations. Moreover, it would be informative to further examine the role of the verbal feedback given during the 6MWT on trait- and state-PF. We also note that the H10 Polar device was previously validated to measure HR and R–R in men and women, but not yet in pwMS.

Our findings demonstrate that trait and state physical fatigue in a wide range of MS severity (EDSS 0–5.5) can be estimated using gait and heart rate measures and that walking bouts of 3 min are sufficient for such estimation. More specifically, minimal double support, cadence under the curve, change in R–R, resting R–R, and TUG-DT are found to be related to physical fatigue. The study suggests these measures as objective markers for trait-PF and state-PF in individuals with MS, laying the foundation for further examination of these measures in other neurological populations. Moreover, the findings of this study lead us to a deeper understanding of the physiological and physical determinants of PF. Future research should also investigate gait and ANS function in free-living conditions to identify daily life markers of physical fatigue. Developing standardized objective markers for assessing and tracking physical fatigue in MS could improve patient management, detect changes over time, and perhaps, more accurately and sensitively measure treatment effects.

### Supplementary Information

Below is the link to the electronic supplementary material.

Supplementary file1 (DOCX 139 kb)

## Data Availability

The data presented in this study are available upon request from the corresponding author and upon consideration of human studies and Helsinki approvals.

## Declarations

### Standard protocol approvals, registrations, and patient consents

Institutional review boards and ethical committees of Tel-Aviv Sourasky Medical Center, Israel approved the study (0959-20-TLV). All patients provided written informed consent before any study procedure was performed.
