# Supplementary material for: Gait and heart rate: do they measure trait or state physical fatigue in people with multiple sclerosis?
Source: J Neurol. 2024 May 2;271(7):4462–72. doi: 10.1007/s00415-024-12339-8 (PMC11233359; doi:10.1007/s00415-024-12339-8)
Supplement: Supplementary file 1 — Supplementary file1 (DOCX 139 kb) [file 415_2024_12339_MOESM1_ESM.docx]

**Supplementary Methods**

Explanation of cadence area measure

As shown in Supplementary Figure 1 (below), the cadence area represents the AUC, the area under the curve, of cadence during the minutes of the 6MWT test.

*The area under a curve between two points is determined by doing a definite integral between the two points. To find the area under the curve y = f(x) between x = 1^st^ min & x = 3^rd^ min or between x = 1^st^ min & x = 6^th^ min, integrate y = f(x) between the limits of a and b. This area can be calculated using integration with given limits.*

Our cadence area estimation included normalization of the original values (right y-axis of in Supplementary Figure 1) within the margins of min and max. The values on the left y-axis are the normalized values. Then, we performed interpolation (+100 points between each two (minute) points, and finally, we calculated the calmative sum of the interpolated points to estimate the cadence area.

*
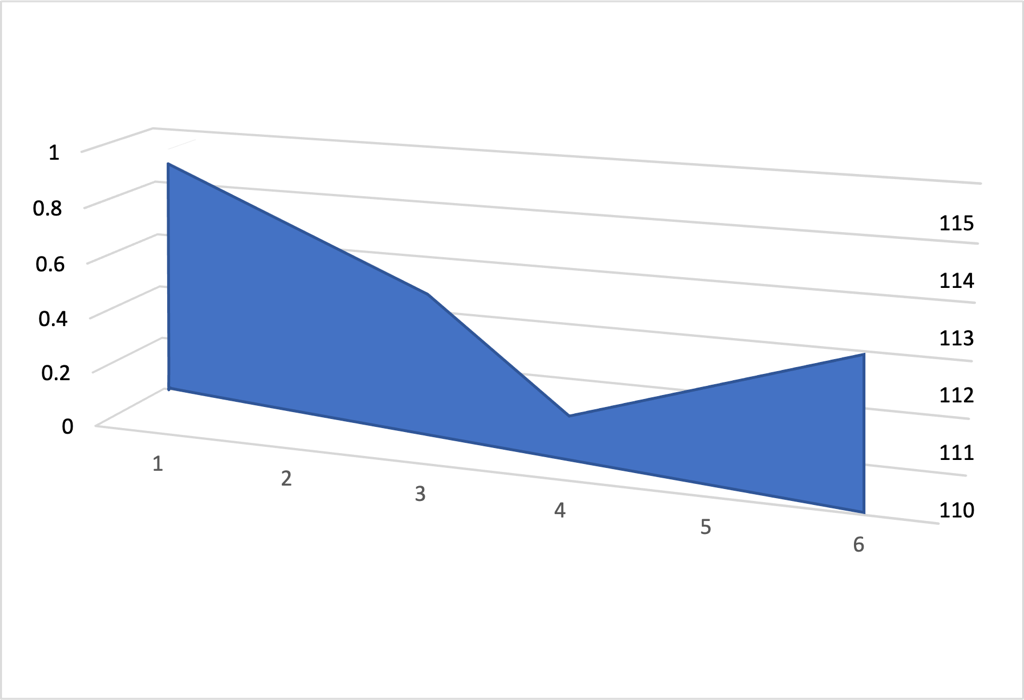
*

**AUC**

Cadence (Steps/min)

Normalized values

**Supplementary Figure 1:** Mean cadence area of the cohort. The cadence area mean value for 6min was 39.5 [95% CI 31.23-47.10] and for 3min was 51.3 [95% CI 43.6-59.0]. AUC- area under the curve.

min

**Supplementary Results**

**Supplementary Table 1:** List of prescribed medication

|  | | | **N** | **%** |
| --- | --- | --- | --- | --- |
|  |  | **MS - related** | 42 | 87.5 |
| **Medication group** | **DMT** | Interferons/Copaxone | 2 | 0.4 |
|  |  | Natalizumab | 7 | 14.5 |
|  |  | S1P Modulators | 6 | 12.5 |
|  |  | B cell depletion | 6 | 12.5 |
|  |  | Fumarate | 12 | 25.0 |
|  |  | Cladribine | 6 | 12.5 |
|  |  | Teriflunomide | 2 | 0.4 |
|  | **ST** | Fampridine | 4 | 0.8 |
|  |  | Muscle relaxants | 2 | 0.4 |
|  |  | Cannabis | 8 | 16.6 |
|  |  | Amphetamines | 8 | 16.6 |
|  |  | **Cardiovascular** | 4 | 0.8 |
|  |  | Rate/Rhythm control | 3 | 0.6 |
|  |  | Blood Pressure control | 2 | 0.4 |
| N= Number of subjects receiving meditation within each drug group. %- percent of subjects out of 48 who received the medication.  MS – Multiple Sclerosis. DMT-Disease Modifying Treatment  ST -Symptomatic Treatment | | | | |

**Supplementary Table 2** Minute-to-minute changes in gait and ANS measures

| Gait domain and measure | Performance per minute | | | | | | Main time effect | 1^st^ -3^rd^ min effect | 1^st^ -6^th^ min effect |
| --- | --- | --- | --- | --- | --- | --- | --- | --- | --- |
|  | Min 1 | Min 2 | Min 3 | Min 4 | Min 5 | Min 6 |  |  |  |
| ANS |  |  |  |  |  |  |  |  |  |
| **Heart Rate** [bpm] | 98.74 (12.98) | 104.74 (15.87) | 106.06 (16.10) | 107.37 (17.74) | 108.88 (16.76) | 110.15 (18.65) | **<0.001** | **<0.001** | **<0.001** |
| **R-R intervals** [ms] | 624.83 (88.47) | 591.48 (101.31) | 584.60 (102.080) | 579.62 (108.08) | 569.99 (97.340) | 565.13 (100.69) | **<0.001*** | **<0.001**** | **<0.001**** |
| **SDRR** [ms] | 73.72  (77.97) | 42.53 (133.50) | 39.02 (125.37) | 27.25  (49.05) | 45.05 (102.53) | 50.90 (201.30) | >0.05* | >0.05** | >0.05** |
| Gait |  |  |  |  |  |  |  |  |  |
| **Gait speed** [m/sec]  Pace domain | 1.31 (0.31) | 1.29 (0.31) | 1.27 (0.31) | 1.28 (0.32) | 1.28 (0.34) | 1.31 (0.36) | **0.012** | **0.002** | 0.980 |
| **Cadence** [steps/min]  Rhythm domain | 115.08 (15.58) | 114.58 (15.54) | 113.55 (15.42) | 113.15 (16.48) | 113.08 (16.96) | 113.35 (16.59) | **0.011** | 0.159 | 0.223 |
| **Stride regularity** [%]  Variability domain | 0.69 (0.17) | 0.72 (0.16) | 0.72 (0.16) | 0.70 (0.18) | 0.68 (0.17) | 0.67 (0.18) | **<0.001*** | **0.028**** | 0.341** |
| **Double Support** [%]  Postural stability | 26.61 (0.8) | 26.54 (0.7) | 26.58 (0.8 | 26.57 (0.8) | 26.54 (0.7) | 26.52 (0.8) | 0.462 | 0.996 | 0.942 |
| **Gait asymmetry** [-]  Asymmetry domain | 5.96 (7.80) | 5.98 (7.61) | 5.81 (7.47) | 5.84 (7.42) | 5.75 (7.41) | 5.15 (7.19) | 0.362* | 0.512** | 0.298** |

The average performance per minute is presented as mean (standard deviation). The p-values of the RMANOVA were corrected using the Greenhouse-Geisser method due to violation of the sphericity assumption. Significant effects of the RMANOVA are presented in boldface. An asterisk (*) represents the time effect according to Friedman’s test of repeated measures. A double asterisk (**) denotes the significance of a change in performance as compared to the first minute of the walk according to Wilcoxon Signed Ranks Test, otherwise, the p-value represents significance according to RMANOVA pairwise comparisons. For each measure, the presented post hoc p-values are corrected for multiple comparisons. [-] denotes unitless variable. ANS - autonomic nervous system. HR – heart rate. SDRR – standard divination of R-R.

**Supplementary Table 3** Mean values of the results are presented in Figure 3.

|  | **gait** | mean (s.d.) | **ANS** | mean (s.d.) |
| --- | --- | --- | --- | --- |
| **3 min** | max speed (m/s) | 1.32 (0.33) | max HR (bpm) | 106.30 (15.85) |
|  | max cadence (steps/min) | 115.82 (15.23) | min R-R (ms) | 577.19 (91.04) |
|  | min double Support (%) | 26.42 (5.4) |  |  |
|  | range speed (m/s) | 0.077 (0.50) | % change HR (bpm) | 0.43 (0.22) |
|  | range cadence (steps/min) | 4.32 (4.17) | % change R-R (ms) | 0.44 (0.21) |
|  | range double Support (%) | 0.22 (0.31) |  |  |
| **6 min** | max speed (m/s) | 1.35 (0.35) | max HR (bpm) | 111.03 (18.93) |
|  | max cadence (steps/min) | 116.77 (15.55) | min R-R (ms) | 556.22 (95.75) |
|  | min double Support (%) | 26.32 (5.27) |  |  |
|  | range speed (m/s) | 0.13 (0.07) | % change HR (bpm) | 0.51 (0.27) |
|  | range cadence (steps/min) | 7.83 (8.50) | % change R-R (ms) | 0.51 (0.26) |
|  | range double Support (%) | 0.41 (0.70) |  |  |
| **Baseline** | HR (bpm) | 74.22 (11.07) |  |  |
|  | R-R (ms) | 827.15 (128.07) |  |  |
|  | SDRR (ms) | 54.14 (35.60) |  |  |
|  | TUG (sec) | 9.55 (2.69) |  |  |
|  | TUG-DT (sec) | 10.77 (2.93) |  |  |

Max- maximal. Min – minimal. TUG – timed Up &Go test. TUG-DT – TUG while performing a dual task, serial 3 subtractions.

HR – heart rate. R-R – r-r intervals. SDRR – standard divination of R-R.

**Supplementary Table 4** p-values of the rho correlations presented in Figure 3.

|  |  |  | **max speed (m/s)** | **max cadence (steps/min)** | **min double Support (%)** | **max HR (bpm)** | **min R-R (ms)** |  |  | **range speed (m/s)** | **range cadence (steps/min)** | **range double Support (%)** | **% change HR (bpm)** | **% change R-R (ms)** |
| --- | --- | --- | --- | --- | --- | --- | --- | --- | --- | --- | --- | --- | --- | --- |
| **trait PF** | **3 min** | **A** | 0.002 | 0.007 | >0.001 | 0.003 | 0.008 |  | **C** | 0.625 | 0.738 | 0.225 | 0.074 | 0.081 |
| **state PF** |  |  | 0.005 | 0.030 | 0.014 | 0.423 | 0.373 |  |  | 0.129 | 0.899 | 0.720 | 0.002 | 0.002 |
|  |  |  |  |  |  |  |  |  |  |  |  |  |  |  |
|  |  |  | **max speed (m/s)** | **max cadence (steps/min)** | **min double Support (%)** | **max HR (bpm)** | **min R-R (ms)** |  |  | **range speed (m/s)** | **range cadence (steps/min)** | **range double Support (%)** | **% change HR (bpm)** | **% change R-R (ms)** |
| **trait PF** | **6 min** | **B** | 0.001 | 0.004 | >0.001 | 0.000 | 0.001 |  | **D** | 0.580 | 0.179 | 0.022 | 0.030 | 0.034 |
| **state PF** |  |  | 0.003 | 0.029 | 0.018 | 0.410 | 0.335 |  |  | 0.700 | 0.030 | 0.186 | 0.002 | 0.002 |
|  |  |  |  |  |  |  |  |  |  |  |  |  |  |  |
|  | **baseline** |  | **HR (bpm)** | **R-R (ms)** | **SDRR (ms)** | **TUG (sec)** | **TUG-DT (sec)** |  |  |  |  |  |  |  |
| **trait PF** |  | **E** | 0.424 | 0.416 | 0.939 | 0.001 | 0.021 |  |  |  |  |  |  |  |
| **state PF** |  |  | 0.013 | 0.012 | 0.009 | 0.001 | >0.001 |  |  |  |  |  |  |  |

The p-values of the rho correlations between trait PF, and state PF and **A**: min and max metrics measured during the first 3 minutes of the 6MWT **B**: min and max metrics measured during the entire 6MWT **C**: range and % change of metrics measured during the first 3 minutes of the 6MWT **D**: range and % change of metrics measured during the entire 6MWT **E**: metrics at baseline (before performing the 6MWT). *Statistical significance of p-value<0.05. HR-Heart Rate (bpm), R-R- r-r intervals (ms). SDRR– the standard deviation of R-R, a representative of heart rate variability (ms). TUG- Timed up and go test, TUG-DT – TUG while performing a dual task, serial 3 subtractions.

**Supplementary Table 5** trait-PF explained by baseline metrics.

| **Model** | | | **R2** | **Variables** | | **St. beta** | | **P-value** | |
| --- | --- | --- | --- | --- | --- | --- | --- | --- | --- |
| 1 | .180** | | | EDSS | .425 | | 0.001 | |  |
| 2 | .217* | | | Age,  gender  height  weight  EDSS | .172  .150  .004  -.049  .411 | | 0.230  0.463  0.985  0.771  0.005 | |  |
| 3 | | X | | HR rest | | NA | | NS | |
| 4 | | X | | R-R rest | | NA | | NS | |
| 5 | | X | | TUG | | NA | | NS | |
| 6 | | X | | TUG-DT | | NA | | NS | |

*p-value of the model <0.05**p-value of the model <0.001 ^$^including model #2, X variable did not contribute to the model.

EDSS -Expanded Disability Status Scale. TUG – timed Up &Go test. TUG-DT – TUG while performing a dual task, serial 3 subtractions.

HR – heart rate. R-R – r-r intervals. HR, R-R rest (mean of 2 last min (out of 5) of rest sited, before performing the 6-minute walk).

**Supplementary Table 6** state-PF explained by baseline metrics.

| **Model** | | **R2** | **Variables** | **St. beta** | **P-value** | |
| --- | --- | --- | --- | --- | --- | --- |
| 1 | .111* | | EDSS | .334 | 0.010 |  |
| 2 | .167 | | Age,  gender  height  weight  EDSS | .106  .079  -.286  .136  .317 | 0.457  0.434  0.163  0.411  0.024 |  |
| 3 | .294 ^$^* | | HR rest | .319 | 0.021 | |
| 4 | .298^$^* | | R-R rest | -.342 | 0.016 | |
| 5 | .236^$^* | | TUG | .391 | 0.011 | |
| 6 | .241^$^* | | TUG-DT | .401 | 0.026 | |
| 7 | .391^$^* | | TUG-DT,  R-R rest | .420  -.329 | 0.016  0.018 | |

*p-value of the model <0.05**p-value of the model <0.001 ^$^including model #2. EDSS -Expanded Disability Status Scale.

TUG – timed Up &Go test. TUG-DT – TUG while performing a dual task, serial 3 subtractions. HR – heart rate. R-R – r-r intervals.

HR, R-R rest (mean of 2 last min (out of 5) of rest sited, before performing the 6-minute walk).

**Supplementary Table 7:** trait-PF explained by objective metrics of gait and ANS collected during the first 3 minutes of the 6MWT

| **Models of 3 first minutes** | **R2** | **Variables** | **St. beta** | **P-value** |
| --- | --- | --- | --- | --- |
| 1 | .259* | Max speed/ cadence | .380 | 0.012 |
| 2 | .230* | Candence Area | .334 | .013 |
| 3 | .356^$^* | Max HR | -.301 | .028 |
| 4 | .347** | Min Double Support (%) | .539 | <0.001 |

*p-value of the model <0.05 **p-value of the model <0.001**.** All models included age, gender, weight, height and EDSS as first block. HR – heart rate.

**Supplementary Table 8:** trait-PF explained by objective metrics of gait and ANS collected during all minutes of the 6MWT

| **Models of 6 minutes** | **R2** | **Variables** | **St. beta** | **P-value** |
| --- | --- | --- | --- | --- |
| 1 | X | Min/Max R-R | NA | NS |
| 2 | .273** | Max HR | -.371 | 0.008 |
| 3 | .352 * | % change in HR*%change in speed | .337 | 0.036 |

*p-value of the model <0.05 **p-value of the model <0.001. All models included age, gender, weight, height and EDSS as first block.

X variable did not contribute to the model. HR – heart rate. R-R – r-r intervals.

**Supplementary Table 9:** state-PF explained by objective metrics of gait and ANS collected during the first 3 minutes of the 6MWT

| **Models of 3 first minutes** | **R2** | **Variables** | **St. beta** | **P-value** |
| --- | --- | --- | --- | --- |
| 1 | .356* | % change in R-R | .417 | 0.003 |
| 2 | .277* | HR range | -.369 | 0.013 |
| 3 | X | Cadence range | NA | NS |
| 4 | .101* | Min Double Support (%) | .318 | 0.029 |
| 5 | .342* | R-R range | -.443 | .001 |

*p-value of the model <0.05 **p-value of the model <0.001. All models included age, gender, weight, height and EDSS as first block.

X variable did not contribute to the model. HR – heart rate. R-R – r-r intervals.

**Supplementary Table 10:** state-PF explained by objective metrics of gait and ANS collected during the 6MWT.

| **Models of 6 minutes** | **R2** | **Variables** | **St. beta** | **P-value** | |
| --- | --- | --- | --- | --- | --- |
| 1 | X | Min Double Support (%) | NA | NS | |
| 2 | X | Cadence range | NA | NS |  |
| 3 | X | HR range | NA | NS | |
| 4 | .395** | % change in R-R (rest to 6 min) | .481 | <0.001 | |

*p-value of the model <0.05 **p-value of the model <0.001. All models included age, gender, weight, height and EDSS as first block.

X variable did not contribute to the model. HR – heart rate. R-R – r-r intervals.
